# Risk distribution across multiple health insurance funds in rural Tanzania

**DOI:** 10.11604/pamj.2014.18.350.3394

**Published:** 2014-08-29

**Authors:** Eunice Nahyuha Chomi, Phares Gamba Mujinja, Ulrika Enemark, Kristian Hansen, Angwara Dennis Kiwara

**Affiliations:** 1 Muhimbili University of Health and Allied Sciences, Dar es Salaam, Tanzania; 2 Aarhus University, Aarhus, Denmark; 3 London School of Hygiene and Tropical Medicine, London, United Kingdom

**Keywords:** Health insurance, risk distribution, risk stratification, risk pool fragmentation, cross-subsidisation

## Abstract

**Introduction:**

Multiple insurance funds serving different population groups may compromise equity due to differential revenue raising capacity and an unequal distribution of high risk members among the funds. This occurs when the funds exist without mechanisms in place to promote income and risk cross-subsidisation across the funds. This paper analyses whether the risk distribution varies across the Community Health Fund (CHF) and National Health Insurance Fund (NHIF) in two districts in Tanzania. Specifically we aim to 1) identify risk factors associated with increased utilisation of health services and 2) compare the distribution of identified risk factors among the CHF, NHIF and non-member households.

**Methods:**

Data was collected from a survey of 695 households. A multivariate logisitic regression model was used to identify risk factors for increased health care utilisation. Chi-square tests were performed to test whether the distribution of identified risk factors varied across the CHF, NHIF and non-member households.

**Results:**

There was a higher concentration of identified risk factors among CHF households compared to those of the NHIF. Non-member households have a similar wealth status to CHF households, but a lower concentration of identified risk factors.

**Conclusion:**

Mechanisms for broader risk spreading and cross-subsidisation across the funds are necessary for the promotion of equity. These include risk equalisation to adjust for differential risk distribution and revenue raising capacity of the funds. Expansion of CHF coverage is equally important, by addressing non-financial barriers to CHF enrolment to encourage wealthy non-members to join, as well as subsidised membership for the poorest.

## Introduction

Health systems that rely on health insurance to finance health care often have multiple insurance funds covering different segments of the population. In low-income countries, this is regarded as the most feasible option for securing universal access to quality and affordable health care services [1–3]. This often implies a variation in the distribution of health risks among the different health insurance funds. The degree to which this distribution varies across insurance funds will have an impact on overall efficiency and equity in the health system [4]. This is especially true when the insurance system is fragmented, lacking opportunity for cross-subsidisation across funds. It has been argued that such fragmentation leads to inefficiency, limits risk pool sizes and expansion of coverage to those who cannot afford to pay for insurance [5–7]. Furthermore, such fragmentation may compromise equity within the health system due to differential revenue raising capacities and differential distribution of health risks to the extent that high risk members are unequally distributed among health insurance funds [3, 8, 9]. Tanzania introduced health insurance in 1996, with the long term goal of achieving universal coverage [10]. The main health insurance schemes currently in operation include the National Health Insurance Fund (NHIF), mandatory for public sector employees; the Community Health Fund (CHF), voluntary and district based for the rural population, with an urban equivalent for the informal population, ‘Tiba Kwa Kadi’ (TIKA) and the Social Health Insurance Benefit (SHIB) for members of the National Social Security Fund (NSSF). In addition there are various private health insurance funds mostly covering the formal sector and micro-insurance schemes covering mostly informal sector workers [11, 12]. The CHF and NHIF are the predominant funds, with coverage reaching about 6.6% and 7.2% respectively of the population in 2009 [13].

The NHIF is a single risk pool covering members across the nation. This factor, together with the mandatory nature of the scheme and income based contributions promotes broad risk spreading and cross-subsidisation from the healthy to the sick, and from the rich to the poor. However this risk sharing is limited to the population covered by NHIF. The CHF comprises multiple risk pools, with risk sharing restricted to each district. This lack of risk sharing across districts limits the degree of risk spreading and cross-subsidisation. Furthermore, the flat rate contributions limit cross-subsidisation from the rich to the poor. The voluntary nature of the CHF also makes it vulnerable to adverse selection, where individuals with high health care needs self select into the fund, resulting in a concentration of less healthy relative to healthy individuals within the fund. The CHF and NHIF operate in parallel within the districts, with no risk sharing across the schemes despite the likelihood that some CHFs will have higher health care needs relative to their revenue bases. The fragmented health insurance system, with no transfers between the risk pools contributes to inequity within the health system and limits the necessary cross-subsidisation for achieving universal coverage. However, beyond the theoretical assumptions outlined above, little is known of the variation in risk distribution across the two funds. Risk distribution in health insurance funds provides information about the influence of individual characteristics on health care costs and determines the health risk profile of the funds. This is based on the assumption that individual characteristics partly determine health care utilisation, which in turn partly determines health care expenditure [5, 14]. These individual characteristics are risk factors, which can be demographic factors such as age, sex and ethnicity [3, 15]; epidemiologic factors such as diagnostic data, self reported health status and socio-economic factors such as marital status, employment status and income [16]. Hence in this paper, risk factors refer to individual characteristics that influence health care utilisation and expenditure. Risk distribution indicates the distribution of risk factors within a health insurance fund and for this paper, refers to the proportion of households with members reporting risk factors among CHF, NHIF and non-member households.

Studies have shown that demographic risk factors have an indirect influence on health care utilisation and expenditure by predisposing individuals to illness. Age has been found to be positively correlated with chronic morbidity while negatively correlated with self reported health status [15, 17–19]. Females are more likely to report chronic morbidity and poor health status, while males report more acute morbidity [18]. Demographic factors can explain 1-2% of the variance in health care expenditure [19–21]. Diagnostic factors are more accurate and explain about 3-4% of the variation in health expenditure [16], but their use is limited by availability of such data, especially in low income countries where medical record keeping is non-electronic and often unreliable. Alternatives to diagnostic factors include self reported health status and self-reported illness, which are subjective measures of health status [22]. Nevertheless, studies have shown that self reported health status and self-reported illness are strong prognostic indicators of mortality and have been shown to accurately predict the need for and use of services [14, 16, 18, 19]. Generally, a lower social status is associated with a low level of income, which limits access to health and education, hence a higher risk of morbidity and mortality and a higher likelihood to need and utilise health services [20, 23–26]. This paper analyses whether the risk distribution varies across the CHF and NHIF in two districts in Tanzania. Specifically we aim to 1) identify risk factors associated with increased utilisation of health services and 2) compare the distribution of identified risk factors among the CHF, NHIF and non-member households. Non-member households have been included since they represent the potential future demand side of CHF; hence analysis of the risk distribution among these households provides an understanding of the potential for resource mobilisation and health care needs of this group. The next section presents the methodology and results are presented in the fourth section. The last section provides the conclusion and recommendations.

## Methods

**Study area:** Data was obtained as part of a larger study from Kongwa and Mpwapwa districts in Tanzania over a period of eight weeks between July and September 2011. The two districts were selected due to their different levels of CHF enrollment, and for convenience in terms of logistics and costs. Kongwa has a total of 63,612 households of which 5,800 (9%) are registered with CHF [27]. Mpwapwa has a total of 78,812 households of which 15,540 (18%) are registered with CHF [27]. Each district has only one district hospital, which provides secondary care services, while all the other facilities provide primary care services.

**Sampling method and Sample size:** In each district multistage sampling was used to select first wards, then villages, followed by hamlets (administrative unit composed of a number of villages) and finally households. For the purposes of this study a household is defined as a person or group of people, related or unrelated, who live together, share a common pot of food and/or who share the same membership card (for CHF) or are dependents of the same principal member (contributing member of NHIF households, usually the head of household or spouse). The study population comprised of all households in the two districts, which met this definition. Due to the low proportion of CHF and NHIF households, stratified sampling was undertaken to ensure sufficient representation of each group. Additionally, due to difficulties in identification of households by membership status from the village household register, households were selected from listings of each membership category as follows: CHF households were randomly selected from the CHF register book kept in the health facilities. This was because health facilities are registration points for CHF. The health facilities were selected based on whether the facility catchment area falls within the selected hamlets. The selection was made from households registered from September 2010 to September 2011, to ensure only current CHF members were included. For NHIF households, a list of all Government institutions in the selected wards or villages was obtained from the District Council, from which all available (at the time of the study) NHIF principal members were selected. Non-member households were randomly selected from the village household register in each of the selected villages. All CHF and NHIF households were omitted from the village register using the list obtained from the facility and District Council respectively before selection of non-member households. The sample size was estimated based on the ability to detect a 25% difference in the proportion of CHF and NHIF using a two-sided test with a power of 80% and a significance level of 5%. We used the proportion of households (50%) with at least one health facility visit reported in a similar study in Burkina Faso [28]. This resulted in a sample size of 243 households per group, totaling 729 households. The final sample size estimated was 766 households after adjusting for non-response of 5%.

**Data collection:** A pre-tested structured questionnaire was administered to all household members. For children below 12 years, a parent or guardian was asked to respond on their behalf. The survey included questions on demographic and socio-economic characteristics, self reported health status, the presence of chronic (illness for 3 months or longer) and acute (illness lasting month or less) illnesses, utilisation of health services, membership status, household ownership of assets and consumer durables. Three return visits were made to households where members were not available for interview during the first visit, resulting in a response rate of 90%, with a sample size of 695 households.

**Variables:** Variables were measured at the household level since CHF membership is based on the household. For the purpose of using a common unit of analysis this was maintained even for NHIF households although membership in this scheme is individual based. Health care utilisation was defined as the number of health facility visit in a household during the past 4 weeks (for acute illnesses) or twelve months (for chronic illnesses) prior to the study. This was operationalised as “0” for households reporting less than the average number of visits for the sample (1 visit) and “1” for households with above average number of visits. Self reported health status was operationalised as the presence of household members reporting fair or poor health status with a value of 1 and 0 otherwise. Self-reported illness was defined as the presence of household members reporting acute or chronic illnesses with a value of 1 for presence and 0 otherwise. Age was defined as the presence of household members in age groups 0-5, 15-49 and 60 and above. These age groups were selected due to their association with higher health care needs [17]. Although the 0-5 and 60 and above age groups are exempted from paying user fees, this policy applies to the users while the costs of providing services still have to be borne by the health facility and indirectly by the scheme. We used an asset index as a proxy for household socio-economic status. The asset index was used to group households into quintiles based on ownership of assets and durable goods. First, Principal Components Analysis (PCA) was used to reduce the large number of asset variables to fewer common underlying dimensions or factors, which could be scored and used to create the wealth index. PCA has been used in studies done in developing countries to develop wealth indices as proxies for income or wealth status owing to the complexities of determining actual income [29, 30]. Education level was operationalised by four categories representing no education, primary education, secondary education and above secondary education. To capture differential household ability to provide for basic needs of members, we included household dependency ratio, calculated as the ratio of number of household members aged below 15 and above 64 to the number of household members aged above 15 and below 64 years old [31]. Households were categorized as having high (>100%), moderate (51-100%) or low dependency ratios (<50%).

**Analysis:** Logistic regression was used to estimate the influence of risk factors on health care utilisation for the whole sample irrespective of insurance status. This was done separately for health care utilisation to capture acute (4 weeks recall period) and chronic illnesses (twelve months recall period). An adjustment was made for clustering effects at the household; hence robust standard errors are reported. Risk factors showing a statistically significant positive relationship with health care utilisation were then used in the risk distribution analysis. Chi-square tests were performed to test whether the distribution of risk factors varies across the CHF, NHIF and non-members.

**Ethical considerations:** Ethical approval was sought from the Research and Ethics Committee of Muhimbili University of Health and Allied Sciences. Informed consent was obtained by asking those who agreed to participate in the study to sign a consent form.

## Results

**General characteristics of households:** We obtained complete information on 695 households. Of these households, 224 are registered with NHIF, 233 with CHF and 238 are non-member households. Fifty percent of NHIF households belong to the highest wealth quintile, while more than 50% of CHF and non-member households belong to the lowest two quintiles (ρ < 0.05). CHF households, with a mean household size of 5.3, were larger than NHIF and non- member households, which both had a mean size of 4.7 members (ρ < 0.05, Table 1).

**Table 1:** Socio-economic and demographic characteristics of study population by membership status, (%)

Variable	Membership status			r-value*
	NHIF n = 224	CHF n = 233	Non-member n = 238	
**Wealth status**				0.000
Lowest	1.3	29.2	31.5	
Second	2.7	29.2	24.4	
Third	10.7	23.6	25.2	
Fourth	34.4	13.7	12.6	
Highest	50.9	4.3	6.3	
Household size				0.002
1-5 members	72.3	56.2	69.3	
6 + members	27.7	43.8	30.7	
**Household dependency ratio**^a^				0.000
None	33.9	6.9	14.3	
< = 50%	30.4	20.6	22.7	
51-100%	24.1	33	28.9	
>100%	11.6	39.5	34.3	
Sex (head)				0.047
Male	81.3	84.1	75.2	
Female	18.8	15.9	24.8	
Education (head)	**n = 222**	**n = 233**	**n = 238**	0.000
No education	1.4	20.7	30.7	
Up to Primary	8.1	71.9	63.9	
Up to–Secondary	27.9	6.5	5	
Above secondary	62.6	0.9	0.4	

* Chi square test

a The ratio of number of household members aged below 15 and above 64 to the number of household members aged above 15 and below 64 years old

**Risk factors associated with health care utilisation:** Table 2 presents the risk factors associated with increased health care utilisation. The odds of above average utilisation are higher for households with members reporting acute, chronic illnesses, poor health status, more than five members and high dependency ratio (ρ < 0.01). Based on the above estimation, the following were relevant risk factors included in the analysis of risk distribution: self-reported health status, self-reported morbidity, age, wealth status, dependency ratio and household size.

**Table 2:** Multivariate logistic regression results of risk factors associated with health care utilisation in households, Kongwa and Mpwapwa, 2011 (N = 692)

Variable	Health care utilisation^c^			
	4 weeks		12 months	
	Odds ratio	Robust Std. Err.	Odds ratio	Robust Std. Err.
**Household reported illness episodes (none**^b^ **)**				
Acute	3.697***	1.315	3.605***	1.309
Chronic	3.940***	1.559	3.791***	1.481
**Wealth Status (lowest**^b^ **)**				
Second	1.300	0.459	1.854**	0.561
Third	1.458	0.645	1.842**	0.561
Fourth	1.084	0.238	1.266	0.474
Highest	0.007	0.262	1.500	0.577
**Household size (>5members**^b^ **)**				
6 + members	2.011***	0.314	4.233***	0.861
**Self reported health status (excellent/very good/good health status**^b^ **)**				
Fair/poor health status	1.930***	0.438	2.195***	0.453
**Education (head, no education**^b^ **)**				
Up to Primary	1.251	0.269	0.728	0.204
Up to–Secondary	1.600	0.603	1.180	0.483
Above secondary	2.048**	0.625	1.550	0.634
**Dependency ratio (none**^b^ **)**				
<50%	4.120***	1.201	4.313***	1.764
51-100%	3.136***	1.113	4.961***	2.061
>100%	4.000***	1.763	4.011***	1.848
**Age-groups**				
0- 5yrs (none b)	2.093***	0.509	0.981	0.212
15-49 yrs (none b)	3.000	2.816	3.833**	2.330
60 + yrs (none b)	1.193	0.290	1.475	0.406

* r < 0.1

** r < 0.05

*** r < 0.01

a The ratio of number of household members aged below 15 and above 64 to the number of household members aged above 15 and below 64 years old

b Base category

c Defined as “0” for households reporting less than the average number of visits for the sample and “1” for households with above average number of visits. Average for 4 weeks recall period is 1 visit and for 12 months recall period is 2 visits.

**Distribution of risk factors among CHF, NHIF and non-member households:** The distribution of risk factors by membership status is presented in Table 3 (Table 1 presents the distribution by wealth status, dependency ratio and household size). A higher proportion of CHF households reported at least one illness episode, reported chronic illness episodes and were more likely to have members with “fair” or “poor” health status compared to NHIF and of non-member households (ρ < 0.05). Fewer NHIF households reported having children aged 0-5 years and elderly members compared to CHF and non-member households (ρ < 0.05).

**Table 3:** Distribution of households with members in high risk age-groups, reporting illness and poor self rated health status by membership status, Kongwa and Mpwapwa (%)

Variable	Membership status			r-value *
	NHIF n = 224	CHF n = 233	Non members n = 238	
**Illness episodes**				0.008
None	18.3	11.2	20.2	
Acute only	52.2	56.7	59.2	
Chronic	29.5	32.2	20.6	
**Self reported health status**				0.001
Excellent/very good/ good health status	45.9	29.2	38.7	
Fair/poor health status	54	71	61.3	
**High risk age groups in household**				
**Children aged 1-5yrs**				0.000
None	66.5	43.8	47.1	
Present	33.5	56.2	52.9	
**15-49 yrs**				0.059
None	4.0	5.6	9.2	
Present	95.9	94.4	90.8	
**Elderly (60 yrs and above)**				0.001
None	92.9	82.0	82.8	
Present	7.1	18.0	17.2	

* Chi square test

## Discussion

This paper sought to highlight the differential risk profiles of CHF and NHIF members by analysing the distribution of risk factors among the two schemes. We first identified self-reported health status, self-reported morbidity, age, wealth status, dependency ratio and household size as risk factors for health care utilisation. The results demonstrate that a higher proportion of CHF households reported the risk factors studied. Furthermore, a higher proportion of CHF households were relatively poorer compared to NHIF households. Analysis of risk distribution among non-member households showed a slightly better risk distribution compared to CHF households. At the same time non-member households had a similar wealth status to those of CHF. The risk distribution among the CHF and NHIF households in this study corroborates findings from other similar studies. A recent study in Tanzania reported a higher proportion of CHF members in the lowest and second wealth quintiles, while a higher proportion of NHIF members belonged to the highest wealth quintile [32]. A study in Germany found that individuals with high health risk factors were concentrated in less wealthier insurance funds than in wealthy funds. This was attributed to health insurance membership being influenced by social class and health status being related to social class [8, 9]. In Colombia [33]. demonstrated that the majority of members of the public fund were less wealthy and high risk compared to those of private funds. This distribution was argued to be influenced by a policy allowing those who could afford to opt out of the public insurance fund for private insurance which effectively creamed off the wealthy and low risk individuals. In Chile where high income earners were allowed to enrol with private insurance funds, it was found that the public funds had a higher proportion of low income and high risk individuals compared to private funds [34].

In line with the studies outlined above, our findings indicate the existence of risk stratification within the health insurance market in the districts, whereby the distribution of health risks varies to the extent that there is a high concentration of individuals with similar health risks within each of the two insurance schemes. This could be explained by the existence of multiple risk pools covering population groups that differ by employment, income and location [3]. Eligibility to the CHF and NHIF is based mainly on employment status and location. The majority of NHIF members are employed in the formal sector, while the majority of CHF members are in the self-employed in the agricultural sector. Employees of the formal sector are often characterised by high education levels and higher income levels compared to those in the agricultural sector [31]. A positive correlation between socio-economic status (measured by the indicators occupation, education and income) and health status has been reported elsewhere [23–26]. The risk distribution between the CHF and NHIF can therefore be viewed as a manifestation of risk stratification inherent in a system with multiple risk pools. The differential risk distribution between the CHF and NHIF has implications for equity within the health system. The high health care needs, illustrated by the risk profile, high dependency ratio and household size, and limited revenue raising capacity of the CHF make it difficult for the scheme to provide access to health care beyond the primary level from government owned health facilities. Furthermore, the relatively lower health care needs and higher revenue base of the NHIF makes it possible for the scheme to provide access to a more comprehensive package of services which includes primary, secondary and tertiary services and access to private facilities for services not available in public facilities. This means that although the two schemes may both be improving access to health care, the quality and quantity of services accessed by their respective members differs. The lower CHF revenue raising capacity then implies access to services is based on ability to pay rather than need.

The variation in the distribution of health risks among health insurance funds is inevitable given the existence of multiple risk pools. It is therefore crucial to establish mechanisms for cross-subsidies across risk pools to address the resulting inequity of access. Countries with multiple health insurance funds serving different population groups often establish mechanisms to compensate for the unequal distribution of high risk groups among the funds by enabling risk and income cross-subsidies across the funds [5, 35]. Fragmentation of the CHF and NHIF risk pools limits the level of risk sharing and pooling of revenues to the extent that it prevents risk and income cross-subsidies across households in the district. The risk distribution among non-member households compared to CHF households highlights two issues. First, it provides an indication of lower expected health care needs in relation to the additional revenue if this group were to join the scheme, implying an increased potential for redistribution from the healthy to the sick. It is also an indication that there may be adverse selection into the scheme. The disinclination of non-member households to join the CHF may have been based on the lower likelihood to need health services. Adverse selection limits the degree of risk spreading that is necessary to provide essential health services to insurance members without increasing premium levels [20, 36]. This drives up overall health care costs within the scheme creating sustainability challenges. Hence, if households are adversely selecting into the CHF, the scheme would be faced with double challenges of risk stratification and adverse selection. Consequently, the likelihood of the CHF enrolling households with high risk members is increased compared to that of the NHIF enrolling members with high risk beneficiaries. The potential for adverse selection into the NHIF exists since dependants are not restricted to immediate family members, hence the principal member may select those perceived to be most likely to need health services. However, the mandatory nature of fund significantly reduces the likelihood. Given the cross-sectional nature of this study, we cannot confidently confirm the existence of adverse selection. Further research of a longitudinal nature will be best suited to serve this purpose.

The general similarity in wealth status between CHF and non-member households provides important insight into the issues of enrolment. For non-member households in the middle to highest wealth quintiles (about 44% of non-member households) paying the annual CHF contribution may not present a significant barrier to enrolment. Non-financial barriers to enrolment such as quality of care, distance to the facility, lack of understanding of the concept of prepayment and lack of trust in authorities have been cited as being more important for wealthier households in a number of studies [37–39]. Increased enrolment is crucial for resource mobilisation and broader risk sharing. Non-poor, non-member households therefore represent a missed opportunity, which underscores the need for efforts to address non-financial barriers to enrolment. Second, this finding reinforces the challenge of exclusion of the poor from the benefits of coverage. The proportion of non-member households in the lowest wealth quintiles represents those excluded from the benefits of coverage. Subsidized membership for these households is crucial to promote equity. Interestingly, the proportion of poor CHF households is higher than non-member households. It is not known whether these households benefitted from exemption or subsidised membership or from assistance from charity organisations or wealthy relatives. Further studies of the enabling factors for enrolment of such households may serve to inform efforts to increase enrolment of excluded groups.

Given the cross-sectional nature of our study, variations in risk distribution over time could not be captured; hence our findings can only serve to highlight differential risk profiles at point in time and cannot confirm trends in risk distribution. Unlike other studies that analysed the risk distribution across the entire population, our study was limited only to the CHF, NHIF and non-members in two districts. Hence the results cannot be generalised to the entire country, rather provide an indication of the effects of fragmented risk pools. However, given the general similarity of characteristics of the formal versus informal sector population our results can still be useful for highlighting the effects of fragmented risk pools.

## Conclusion

The study findings provide lessons for policy makers in low- and middle-income countries where multiple health insurance funds have been established to achieve universal coverage. In particular, addressing the challenges of limited risk sharing and cross-subsidisation across multiple health insurance funds remains crucial for equitable access and financial protection. Reducing the existing fragmentation is essential for providing opportunities for cross-subsidisation across the schemes and promoting equity. In this respect, experience from Latin America [40] and Rwanda [41] in the organisation of financial transfers from a central fund to subsidise funds with greater health needs are promising examples. In addition, the paper has highlighted the potential for additional resource generation that exists among non-member households and provided further impetus to address non-financial barriers to enrolment for middle and high-income groups. Qualitative studies focusing demand and supply side factors will provide policy makers with a deeper understanding of what is required to increase enrolment. At the same time exclusion of the very poor reinforces the need for subsidised membership. To this end in addition to financial transfers across the schemes, other domestic sources of revenue could be used to increase resources available for subsidised membership. Examples can be drawn from Ghana, where VAT and Social Security contributions from the formal sector have been used as a source of funds to subsidise membership for indigent [42]: and Gabon where a 10% levy on mobile phone companies turnover and a 1.5% levy on money transfers outside the country are used to fund health benefits for the low income [43].
